# Applications of Fibronectin in Biomedicine and Cosmetics: A Review

**DOI:** 10.3390/bioengineering12111249

**Published:** 2025-11-15

**Authors:** Yuan Wang, Qirong Zhang, Xiandong Zhou, Dingshan Yang, Lin Xiao, Wenlan Xie, Huaping Zheng, Shuiwei Ye, Chaoqing Deng, Yong Cheng, Peng Shu, Qi Xiang

**Affiliations:** 1 HBN Research Institute and Biological Laboratory, Shenzhen Hujia Technology Co., Ltd., Shenzhen 518000, China; 2 Jinan University Hujia Technology Joint Laboratory, Guangzhou 510000, China; 3 Guangdong Engineering Technology Research Center for Functional Skincare Innovation, Shenzhen Hujia Technology Co., Ltd., Shenzhen 518000, China; 4 Guangzhou Jike Meichuang Co., Ltd., Guangzhou 510000, China; 5 Biopharmaceutical R&D Center of Jinan University, Guangzhou 510000, China

**Keywords:** fibronectin, extracellular matrix, skin barrier repair, regenerative medicine, anti-aging

## Abstract

Fibronectin (FN) is a key mechanoresponsive glycoprotein within the extracellular matrix (ECM) that contributes to the assembly of a dynamic fibrillar network that is important for maintaining tissue structure and mediating cellular signaling. In this review, we delineate the molecular mechanisms underlying FN’s role in barrier restoration, ECM remodeling, and stem cell niche regulation, functions that inform its applications in both regenerative medicine and cosmetic science. In biomedical contexts, FN is recognized as a valuable biomarker for numerous diseases, a promising therapeutic target, and a functional component of biomedical material matrices. FN is involved throughout the skin repair process, making it a physiologically active ingredient for cosmetic anti-aging treatments, alleviating sensitive skin conditions, and enhancing cutaneous immunity. This review also addresses significant translational challenges associated with FN research, including recombinant protein production and rational peptide design, and suggests avenues for future work. Ultimately, studies on FN highlight the complexity of ECM biology and lay the groundwork for innovative approaches to advancing human health and developing new cosmetic treatments.

## 1. Introduction

In the complex domain of biological processes, the extracellular matrix (ECM) does not merely serve as a static scaffold but operates as a dynamic and intricate information network that profoundly influences cell behavior and tissue homeostasis [1,2]. Fibronectin (FN), a large multidomain glycoprotein, plays important roles in numerous biological processes owing to its distinctive molecular structure and functional characteristics. Its cell-adhesive and signaling functions are integral to processes such as cell migration, tissue repair, and immune responses. FN serves as both a structural adhesive and a mechanosensitive signaling center within the ECM [3,4,5].

The complexity of FN’s function is evident not only in its role in maintaining physiological homeostasis—such as embryonic development, wound healing, and tissue repair—but also in its significant association with the pathological progression of major diseases, including tumor metastasis, fibrosis, inflammatory diseases, and cardiovascular disorders [6,7,8,9,10]. Its role as a modulator of cell behavior, combined with disease-specific alterations in its expression or modification, makes it a promising diagnostic biomarker and a potential target for therapeutic intervention [11,12].

Recent advancements in recombinant expression and functionalization technologies have accelerated the translational potential of FN and its derivatives [13,14]. This review seeks to provide a comprehensive analysis of FN, tracing its progression from fundamental molecular structure to advanced biomedical applications. Initially, the discussion will focus on its functional motifs and the diversity of its isoforms, which contribute to its functional versatility. Subsequently, the review will examine the implications of its distinct cellular and plasma-derived forms, as well as its pathological accumulation, emphasizing its role as a biomarker and therapeutic target in various diseases. Furthermore, the intrinsic regenerative and reparative properties of FN will be elucidated to support its applications in regenerative medicine and cosmetic treatments. Given the limitations associated with natural extraction methods, recombinant genetic engineering approaches will be explored as solutions to production challenges and a means to enable functional customization. Finally, we address the ongoing challenges in the field through the lens of this problem-solving framework, proposing future directions that prioritize the quantitative linkage of FN’s mechanisms to tangible clinical benefits. This approach aims to guide the rational development of FN-based therapies and cosmetics from bench to bedside.

## 2. Molecular Structure

### 2.1. Domain Organization and Functional Motifs

The biological functions of FN are governed by specific functional motifs embedded within its modular domain structure. FN is a dimeric glycoprotein, with each monomer having an approximate molecular weight of 250 kDa, typically linked via C-terminal disulfide bonds. Each monomer comprises three distinct types of modules: 12 type I domains (FNI), 2 type II domains (FNII), and 15 to 17 type III domains (FNIII) [4,15]. These domains constitute the structural basis for the functional motifs. A well-characterized example is the integrin-binding Arg-Gly-Asp (RGD) motif located in the FNIII10 domain, which acts synergistically with the adjacent Pro-His-Ser-Arg-Asn (PHSRN) sequence in the FNIII9 domain. The RGD motif mediates interactions with specific integrins, such as α5β1 and αvβ3, thereby facilitating cell adhesion and migration, while the PHSRN sequence enhances the binding affinity for integrin α5β1 [16]. Additional functional motifs include collagen-binding regions primarily situated within the I6-9 and II1-2 domains. Heparin-binding sites, notably within the Heparin II domain, interact with glycosaminoglycans (GAGs) and may contribute to the recruitment of growth factors. Moreover, fibrin-binding regions are located in both the N-terminal (I4-5) and C-terminal (I10-12) domains [17,18]. The FNIII domains display mechanoresponsive characteristics; their β-sandwich structure lacks disulfide bonds, rendering them susceptible to mechanical unfolding, which can expose cryptic binding sites and modulate signaling pathways [4,19].

### 2.2. Isoform Diversity Through Alternative Splicing

The function of FN is further regulated by the alternative splicing of its pre-mRNA, which results in the variable inclusion of isoforms containing the Extra Domain A (EDA^+^), Extra Domain B (EDB^+^), and the V region segments. The EDA domain is implicated in modulating cell migration and inflammatory responses through its interactions with integrins α4β1 and α9β1 [20,21]. The EDB domain has been linked to angiogenesis and exhibits increased expression under certain pathological conditions [22,23,24]. Additionally, the IIICS region contains binding motifs, such as LDV and REDV, that interact with the α4β1 integrin, thereby potentially affecting cell adhesion and migration [4,25,26]. This structural heterogeneity underlies the context-dependent functional roles of FN.

**Figure 1:**
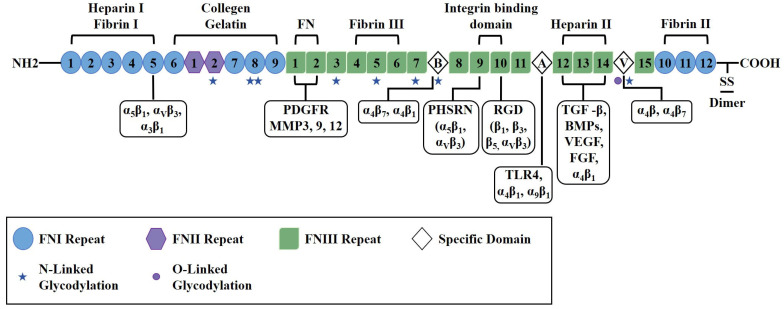
Schematic diagram illustrating the structure of fibronectin, including various domains, receptor binding sites, and glycosylation sites. The individual domains are categorized as Type I (blue circle), Type II (purple hexagon), Type III (green square), or variable region (white prism). Regions and specific sequences demonstrated to bind other ECM constituents are shown above the structure, with Roman numerals denoting the particular type I, II, or III repeats involved. Binding sites for integrins and signaling molecules are shown below the structure. FN contains multiple glycosylation sites, represented by star or round shapes. This diagram is adapted from Patten and Wang, based on the current structural knowledge of fibronectin [18].

FN serves as a multifunctional ligand through its interaction with various ECM components, including integrins, collagen, fibrin, glycosaminoglycans (GAGs), and growth factors (e.g., TGF-β, VEGF) [17]. Post-translational modifications, including glycosylation and phosphorylation, may further influence FN’s functional properties [2,27,28]. The structure of FN and associated binding/glycosylation sites are shown in Figure 1.

## 3. Physiological Forms and Pathological Expressions: Diagnostic and Therapeutic Implications

### 3.1. Plasma and Cellular FN: Distinct Characteristics

FN exists in two primary forms: plasma FN (pFN), which is predominantly synthesized by hepatocytes and plays a role in coagulation and opsonization processes; and cellular FN (cFN), which is locally assembled by fibroblasts, endothelial cells, and other cell types into ECM fibrils [29]. These forms differ in their assembly properties and functional roles, with cFN contributing to tissue architecture and acting as a scaffold for collagen deposition [30].

### 3.2. Altered Expression in Pathological Conditions

Altered expression patterns of specific FN isoforms have been documented in various pathological conditions. For example, increased expression of the EDA-positive FN isoform has been observed in certain tumor microenvironments and has been associated with disease progression in multiple studies [31]. In the context of diabetic vascular complications, hyperglycemia has been linked to FN accumulation, which may contribute to endothelial dysfunction via integrin-mediated signaling pathways [32]. Serum levels of sialylated FN have been identified as a potential prognostic indicator in thyroid cancer [33]. Consequently, alterations in FN expression or post-translational modification patterns may serve as valuable biomarkers for disease diagnosis and prognosis [31,33,34,35].

The consistent disease-associated expression of specific FN isoforms and fragments has prompted investigations into their potential to be investigated as therapeutic targets. Current approaches under consideration include developing agents that target the EDA-FN isoforms, modulating the pathological accumulation of FN, and neutralizing the bioactivity of specific pro-inflammatory FN fragments [36,37,38]. Notably, the overexpression of FN in fibrotic tissues has been exploited for targeted drug delivery. This strategy is exemplified by an inhalable nano-in-micro system functionalized with the FN-binding CREKA peptide, which exhibited enhanced targeting of lung myofibroblasts and improved therapeutic efficacy in pulmonary fibrosis [39].

### 3.3. The Context-Dependent Dual Faces of Fibronectin

The biological activities of FN exhibit considerable variation between physiological and pathological contexts. In disease microenvironments, such as those found in cancer or fibrosis, the altered expression, splicing, and proteolytic processing of FN are recognized features that contribute to the progression of pathological processes [36,40]. Conversely, during tissue repair and regeneration, tightly regulated FN expression facilitates constructive remodeling. This functional duality underpins therapeutic strategies aimed at inhibiting pathological FN functions in disease while promoting its beneficial roles in regeneration. Such insights enable the targeted application of FN in regenerative medicine and cosmetics, where the objective is to enhance its physiological functions in cell adhesion and ECM assembly, alongside the development of approaches to inhibit pathological isoforms, such as EDA^+^, in disease treatment. The subsequent sections examine these distinct translational pathways in greater detail.

## 4. Function Dictates Application Scenarios: Regenerative Medicine and Cosmetic Science

### 4.1. Foundations and Applications in Regenerative Medicine

FN significantly enhances cell migration, proliferation, and angiogenesis, thereby serving as a key functional factor in various dressings, scaffolds, and biomaterials aimed at addressing clinical challenges in tissue repair.

#### 4.1.1. Wound Healing Applications

In the context of acute wounds, delayed healing is associated with an increased risk of infection and, following healing, frequently results in hypertrophic scarring or scar contracture, which adversely affects both esthetic outcomes and joint mobility. In cases of extensive burns or skin graft sites, compromised blood flow impedes granulation tissue formation and reduces the survival rate of skin grafts. Numerous studies in murine full-thickness skin defect models have demonstrated that adding FN or its functionalized fragments (e.g., those modified via click chemistry, nanocarriers, or FN synergy sites) can promote wound closure to a clinically meaningful extent, accelerating it by approximately 2 to 3 days and increasing the healing rate by roughly 20% to 30%. Notably, Irene Gimeno-LLuch et al. reported that surface-modified fibronectin contributes to improved scar quality [41,42,43]. Furthermore, FN applications include accelerating epithelial regeneration in rat corneal abrasion models, promoting wound closure when covalently bound to absorbable sutures, and facilitating the rapid healing of oral ulcers [44,45]. Wenwu Zhang et al. identified that the peptide segment P12, derived from FN, mitigates burn progression and promotes granulation tissue formation by enhancing platelet-derived growth factor BB (PDGF-BB) signaling, reducing apoptosis in burn tissue, and increasing fibroblast survival. In an atopic skin transplantation model, exogenous fibronectin supported an improvement in the wound healing rate by about 30% [46]. Additionally, in a human skin xenograft model of atopic dermatitis, IL-4 treatment suppressed FN expression and delayed wound healing, while topical application of exogenous FN reversed this delay and promoted wound closure, alongside restoration of epidermal differentiation complex gene expression [47]. Despite these promising findings in vitro and in vivo, recombinant human fibronectin (rhFN) products are still mainly in the preclinical research stage.

The challenges of chronic wounds extend beyond a prolonged healing process to include fundamental impairments in repair mechanisms, significantly increasing the risk of severe complications. Statistical data show that approximately 15% to 25% of diabetic patients will develop foot ulcers during their lifetime, and the healing process in these cases is notably complex and protracted. Furthermore, about 20% of cases involving moderate to severe infections ultimately result in lower limb amputation [48,49]. Research by Xiaomin Li et al. demonstrates that the recombinant protein rhFEB, which integrates EDB-FN and COL3A1, can construct a scaffold structure similar to the natural extracellular matrix in diabetic mice. This scaffold significantly enhances cell adhesion and migration, promotes angiogenesis, and thereby remodels the wound microenvironment, leading to a demonstrable improvement of the wound healing process by day 14 [50]. Similarly, a composite dressing developed by Táborská et al. has been shown to expedite wound closure in a diabetic foot ulcer model [51]. Additionally, Jianhang Chong et al. constructed a rhFN1024 hydrogel system carrying periodontal ligament stem cells, which facilitates cell survival, angiogenesis, and collagen deposition in full-thickness skin wounds of diabetic rats through activation of the NF-κB signaling pathway. Notably, three days post-injury, the wound healing rate in the hPDLSCs@H-rhFN_1024_ group reached 62.7% ± 3.63%, which was 1.8-fold higher than the control group [52]. These findings hold considerable promise for clinical application, with the potential to convert chronic, non-healing wounds into actively healing states. The ultimate clinical value, however, will be determined by the ability of such FN-based therapies to achieve a defined, acceptable reduction in amputation and recurrence rates within the target diabetic population, thereby addressing the core unmet need. Nonetheless, the extent to which these therapeutic strategies can ultimately translate into a clinically acceptable reduction in patient amputation rates remains to be confirmed through rigorous clinical trials.

The clinical application of autologous platelet-rich fibrin (PRF) serves as a practical counterpart to preclinical strategies and has demonstrated considerable efficacy in the management of chronic wounds. PRF is a naturally formed fibrin matrix that functions as an optimal physiological scaffold, richly incorporating endogenous fibronectin, growth factors, and immune cells. Its therapeutic effectiveness reflects the biological roles of fibronectin—such as cell adhesion, migration, and extracellular matrix remodeling—translated into an accessible and cost-effective clinical intervention. A retrospective analysis involving 105 patients with various refractory wounds, including diabetic foot ulcers, vascular ulcers, and pressure injuries, reported an overall effective rate of 97.1% following PRF treatment, with 92 patients achieving satisfactory healing [53]. Further robust evidence is provided by a randomized controlled trial, which demonstrated that PRF therapy significantly outperformed conventional wound care. Patients treated with PRF exhibited a more substantial reduction in pro-inflammatory cytokines (TNF-α, IL-2, IL-8), higher wound healing rates, shorter healing durations (26.54 ± 6.95 days versus 32.41 ± 6.87 days), lower pain scores, enhanced quality of life, and decreased treatment costs [54]. The well-documented clinical success of PRF highlights the therapeutic potential of delivering a fibronectin-rich microenvironment to chronic wounds, thereby effectively promoting the transition from a non-healing to a healing state.

#### 4.1.2. Role as a Biomedical Material Matrix

A major challenge in regenerative medicine is the limited delivery efficiency of bioactive molecules, including growth factors. FN, due to its intrinsic capacity to bind and stabilize these molecules, has emerged as a key component in the development of delivery systems designed to overcome key clinical limitations. For example, incorporating FN into hydrogel carriers, such as PEG-FN, markedly improves the retention and localized presentation of growth factors like bone morphogenetic protein-2 (BMP-2). In animal models of bone defects, this strategy has been shown to enhance bone formation compared to the application of BMP-2 alone [55,56]. These findings suggest that FN-enriched matrices can effectively address the challenges of rapid diffusion and short half-life associated with growth factor therapies, thereby representing a promising strategy for achieving functional bone regeneration in complex defects.

Beyond its role in growth factor delivery, FN and its engineered constructs exhibit considerable potential in musculoskeletal repair. The depletion of FN within the aged muscle extracellular matrix has been implicated in impaired satellite cell function and compromised regenerative capacity. In this regard, a computationally designed recombinant FN construct (rhFN-NM) has been shown to enhance myoblast adhesion, viability, and resistance to oxidative stress in vitro. Furthermore, in vivo administration of rhFN-NM in aged mice facilitated muscle regeneration by modulating the inflammatory microenvironment, promoting M2 macrophage polarization, and improving stem cell function [57]. These findings underscore the potential of FN-based biologics to actively regulate the regenerative niche in musculoskeletal tissues, extending beyond their traditional role as passive scaffolds.

In the domain of implantology, the long-term functionality of implants is frequently limited by the host’s foreign body response and insufficient integration with surrounding tissues. Surface coatings modified with FN or its functional peptide sequences, such as the RGD motif, are problem-driven designs aimed at actively promoting the adhesion and functionality of specific target cell types [58,59]. For example, the FN-heparin composite coating is designed to concurrently address two primary challenges associated with blood-contacting implants: thrombosis and delayed endothelialization [60]. By integrating the cell-adhesive properties of FN with the anticoagulant effects of heparin, this coating aims to facilitate the development of a stable endothelial layer, thereby improving the implant’s long-term biocompatibility. Although this dual-targeting approach is conceptually sound, its ability to prevent implant failure and meet long-term safety and efficacy standards requires validation through extended in vivo studies and, ultimately, confirmation in human clinical trials.

### 4.2. Cosmetic Applications: Skin Repair and Anti-Aging

In light of the relevant research in the biomedical field, FN has been increasingly applied in the cosmetics market and has gradually gained recognition among consumers [61].

#### 4.2.1. Barrier Repair and Anti-Sensitivity

Sensitive skin is a common skin problem, with approximately 60% to 70% of women and 50% to 60% of men who report varying degrees of skin sensitivity [62]. The primary clinical features include facial skin redness, dryness, increased fine lines, and a thinning of the stratum corneum, all of which are closely associated with compromised skin barrier function. Consequently, simple hydration alone is insufficient to effectively address these dermatological concerns. Loricrin (LOR), a key structural protein of keratinocyte envelopes, and filaggrin (FLG), whose metabolites serve as natural moisturizing factors, collaboratively maintain skin barrier integrity, contribute to the formation and hydration of the stratum corneum, and play a critical role in reducing transepidermal water loss (TEWL) [63].

Yuan Wang et al. reported that a recombinant human fibronectin peptide (rhFNP), derived from the extracellular matrix, could promote the expression of LOR in normal human epidermal keratinocytes (NHEK) and increase the transcription levels of FLG and LOR in mouse skin [64]. Zou Jie et al. conducted a four-week study on rhFN products in individuals with damaged scalps, observing a substantial 24.2% reduction in TEWL, which indicates a meaningful improvement in skin barrier integrity of skin barrier integrity [65]. Zhouqiao Xie et al. demonstrated that overexpression of FN effectively reverses epithelial barrier damage caused by excessive degradation, such as that mediated by matrix metalloproteinase 12, thereby promoting barrier function recovery [66]. Additionally, Yunqing Dong et al. showed that the combined application of rhFN and recombinant human collagen (RHC) significantly inhibits inflammatory responses. Compared to the control group, the expression levels of inflammatory factors TNF-α, IL-1β, and IL-6 decreased by 42%, 45%, and 38%, respectively, in the co-treatment group, indicating a significant anti-inflammatory effect [67]. Several cosmetic companies have launched essence, face cream, and scalp care products with recombinant FN peptide as the core ingredient. In summary, fibronectin not only maintains the integrity and stability of the skin’s structural barrier but also exerts anti-inflammatory effects, making it of great significance in the repair of sensitive skin.

#### 4.2.2. Anti-Aging Applications of FN

FN is also being explored for its potential to address features of skin aging, such as wrinkles, loss of elasticity, and thinning, which result from ECM degradation and decreased cellular function.

At the ECM level, the CBD and HepII domains of FN are thought to contribute to the stabilization of collagen fibers and suppression of MMP activity, thereby potentially reducing collagen degradation [56]. FN’s antioxidant and anti-glycation properties may also inhibit the pathological cross-linking of collagen that contributes to loss of resilience [68]. Preclinical studies have shown that recombinant FN peptides can enhance dermal collagen content in photoaging models, indicating a potential role in ECM remodeling [69].

At the cellular level, FN appears to promote fibroblast synthesis of collagen and elastin through integrin-mediated signaling (e.g., α5β1/FAK/PI3K/Akt) and may support mitochondrial function [70,71,72]. It also helps maintain the stemness of epidermal stem cells and delays the senescence of adipose-derived stem cells (ADSCs) by reducing the expression of senescence-associated genes and inhibiting the SASP [73,74,75]. This suggests a potential role for FN in mitigating the accumulation of senescent cells that disrupt the tissue microenvironment.

**Figure 2:**
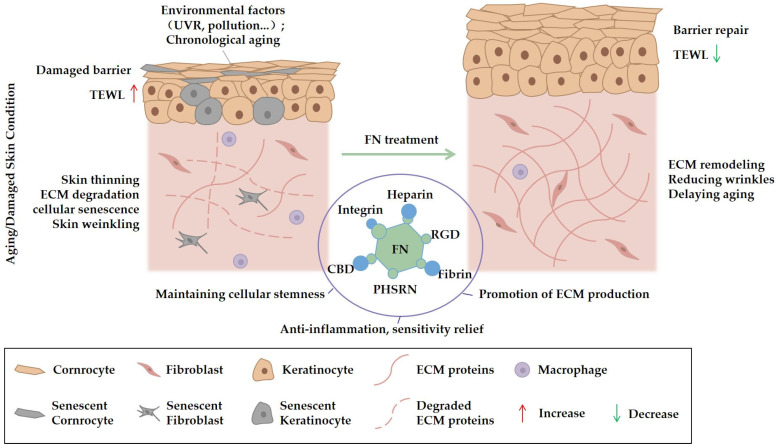
This schematic diagram depicts the mechanism by which fibronectin (FN) ameliorates skin aging and damage. The left panel illustrates skin compromised by environmental factors, including ultraviolet radiation and pollution, as well as intrinsic chronological aging. Manifestations of damage include barrier impairment, increased TEWL, epidermal thinning, ECM degradation, cellular senescence, and wrinkle formation. Following FN treatment (right panel), restoration of the skin barrier and reduction in TEWL are observed. Additionally, FN contributes to the maintenance of cellular stemness, stimulation of ECM synthesis, anti-inflammatory effects, and alleviation of sensitivity. These functions are mediated through FN’s structural domains, such as the heparin-binding domain, integrin-binding domain (containing the RGD sequence), fibrin-binding domain, PHSRN sequence, and collagen-binding domain (CBD). Collectively, these activities facilitate ECM remodeling, wrinkle attenuation, and delay of the aging process.

In summary, preclinical evidence indicates that FN can influence several key pathways involved in skin aging. By potentially stabilizing the ECM, supporting cellular synthetic capacity, and mitigating aspects of cellular senescence, FN represents a promising candidate worthy of further research for delivering improvements in the visible signs of aging (Figure 2).

## 5. Recombinant Production Approaches: Addressing Manufacturing Challenges

### 5.1. Production Advantages and Technical Optimization

The conventional methods for extracting FN from plasma or animal tissues are associated with several significant limitations, including low yield, risk of pathogen contamination, potential immunogenicity, procedural complexity, poor stability, susceptibility to inactivation, and unpleasant odors. Plasma-derived FN has a distinct odor, limiting its use in consumer skincare products due to low acceptance. Collectively, these factors restrict the practical application of these types of extraction methods. Recombinant expression technology addresses these limitations by enabling safe and efficient large-scale production of proteins in host cells, such as E. coli and yeast. The ongoing optimization of the expression vectors and cultivation techniques used in these processes has substantially enhanced production yields [4,76,77]. Utilizing expression systems such as yeast guarantees appropriate glycosylation, which is essential for functional activity [78]. Recombinant FN also avoids the odor problem, improving suitability for consumer products.

### 5.2. Methods for Detecting Recombinant Fibronectin in Cosmetics

Using recombinant FN (rFN) in cosmetic formulations necessitates the development of precise quantification methods to ensure effective quality control; this is challenging due to interference from complex cosmetic matrices and potential conformational changes. The detection techniques for rFN can be broadly classified into two categories: qualitative or structural detection and content quantification. The characteristics, applications, advantages, and disadvantages of each method are summarized in Table 1. Furthermore, bioactivity assays, such as cell adhesion and migration tests, can provide indirect evaluations of functional activity. While these assays are valuable for research and development, they are inadequate for the accurate quantification required for clinical/cosmetic applications.

### 5.3. Expanding Applications and Innovations of Recombinant Fibronectin in Skin Repair

The progress in recombinant fibronectin (rFN) technology has significantly expanded its potential application scope. Epidermal growth factor (EGF) is widely used in skin repair due to its strong mitogenic effect on keratinocytes and fibroblasts. However, the use of EGF in Chinese cosmetics has been banned due to regulatory restrictions, the potential tumor risk from long-term use, and insufficient formula stability. In contrast, recombinant fibronectin, as an endogenous ECM component, demonstrates higher safety with its multiple repair mechanisms, effectively overcoming the challenges of safety, regulatory compliance, and stability mentioned above, and thus can be an alternative to EGF [86]. Additionally, recombinant fibronectin peptides have relatively lenient requirements for storage conditions, which helps simplify formula design and extend the product’s shelf life.

## 6. Discussion

This review systematically delineates the extensive application prospects of FN in domains such as regenerative medicine and cosmetic science. This transition is substantiated by concrete outcomes, notably the initial regulatory approval of an FN-based drug in India in 2020, followed by its commercialization in 2022. Nevertheless, the central challenge confronting current research is how to effectively translate the remarkable biological activities of FN manifested in preclinical models into practical clinical and consumer—level solutions that meet acceptable thresholds for both efficacy and safety. Future efforts should shift from a molecule-driven to a problem-driven paradigm. That is, the initial step is to precisely define the unmet clinical needs in terms of the minimum acceptable level of functional recovery and the maximum tolerable risk of adverse events, followed by rationally devising FN—based intervention strategies and establishing a causal linkage from molecular mechanisms to these clinically acceptable outcomes.

In the realm of regenerative medicine, adhering to the “problem—driven” tenet, the value of FN should be gauged by its capacity to enable therapies that meet the acceptable clinical standards for specific indications. Regarding chronic wounds like diabetic foot ulcers, the unmet clinical requirements extend beyond merely “accelerating wound healing.” The emphasis is placed on attaining a clinically acceptable rate of wound closure and restoring a functionally intact skin barrier. This necessitates the reversal of the pathological microenvironment and the construction of an ECM scaffold that promotes tissue regeneration. The primary clinical objective is to achieve a specified and acceptable reduction in amputation and recurrence rates within the target population. In the context of implant applications, the core objective of FN—functionalized coatings should be centered on rectifying the mismatch between the host and the implant. This can be achieved by facilitating a level of endothelialization sufficient to prevent thrombus formation or directly promoting a degree of osseointegration that prevents implant loosening, ultimately attaining the clinical endpoint of long—term implant functionality with an acceptably low incidence of adverse events.

Within the domain of cosmetic science, the central issue is that numerous products can only effect transient improvements and struggle to deliver a sustained, clinically measurable repair of skin barrier defects and structural aging. Consequently, FN should be positioned as an active repair agent targeting the root pathological causes. The evaluation of its efficacy should not solely rely on short—term enhancements in sensory perception but should be substantiated by objective instrumental data demonstrating that key parameters (e.g., TEWL, elasticity) are maintained within a range indicative of a healthy, resilient skin condition over the long term. This includes demonstrating its ability to achieve a long—term restoration of barrier resilience (such as a sustained reduction in transepidermal water loss to levels comparable to undamaged skin) and a quantitative reconstruction of ECM structure (such as an increase in collagen density towards that of younger skin). This implies that the research and development of FN—related products must transcend mere descriptive accounts of surface phenomena and delve into elucidating how specific structural domains (such as RGD and HepII) modulate pivotal signaling pathways, thereby promoting long-term skin homeostasis and resilience.

Despite the promising prospects of recombinant FN in regenerative medicine and cosmetics, the challenges encountered during its industrialization remain a crucial bottleneck in realizing its clinical translational value. Currently, the industrial production of recombinant FN is constrained by multiple factors, including insufficient yields from expression systems, intricate downstream purification processes (notably the high—cost chromatography steps), and the difficulty of effectively controlling the stability of recombinant proteins. These factors jointly contribute to a substantial increase in the cost of the final product, which not only limits its large-scale application in biomedical materials but also erects a significant price barrier to its market penetration in the cosmetics industry. Therefore, future research endeavors should not only continue to deepen the mechanistic understanding and engineer FN variants that reliably meet clinical acceptability criteria but also accord equal strategic importance to process innovation and cost control. The development of efficient and cost-effective novel expression systems and purification technologies is essential for producing FN-based products that are both biologically active and economically viable, thereby ensuring their compliance with cost–benefit criteria necessary for widespread clinical and consumer adoption.

## 7. Conclusions

Translating the complex biological functions of FN into effective clinical and cosmetic applications necessitates a fundamental paradigm shift. Future research should move from a technology-driven to a “problem-driven” strategy, prioritizing the achievement of defined clinical acceptability thresholds rather than solely optimizing performance metrics. This approach requires the precise delineation of unmet needs, characterized by the minimum acceptable level of benefit (e.g., a targeted reduction in amputation rates) and the maximum tolerable risk, followed by the development of FN-based interventions designed to consistently meet these criteria. Simultaneously, addressing the industrial challenges associated with recombinant FN production is essential, with an emphasis on attaining acceptable yields, purity, and cost-effectiveness, rather than merely overcoming technical obstacles. By adopting this disciplined, constraint-focused framework, FN research can progress from demonstrating biological potential to delivering solutions with predictable and acceptable impacts on human health.

## Figures and Tables

**Table 1:** Common methods for detecting recombinant fibronectin in cosmetics.

Purpose	Method	Primary Use/Characteristics	Advantages	Disadvantages/Limitations	References
Qualitative/ Structural Detection	Electrophoresis (SDS-PAGE)	Molecular weight and purity	Simple, provides basic information	Lacks specificity, not quantitative	[79]
	Western blot (WB)	Identity using specific antibodies	High specificity, confirms target protein	Semi-quantitative, multi-step procedure	[66]
	Infrared Spectroscopy (IR)	Provides secondary structure information	Useful for structural analysis	Susceptible to interference, hard to quantify	[80]
	Peptide Mapping (e.g., LC-MS/MS)	High-precision identification via characteristic peptides after enzymatic digestion	High accuracy and specificity	Expensive instrumentation, complex workflow	[81]
	Amino Acid Analysis	Indirect absolute quantification via acid hydrolysis	Absolute quantification, high accuracy	Time-consuming, requires expertise	[82]
Content Detection	Total Protein Assays (e.g., BCA)	Estimation of total protein content	Fast, economical, high-throughput	Lacks specificity, prone to interference	[83]
	Immunoassays (ELISA)	Highly sensitive and specific quantification using antibodies	High sensitivity, specific, widely used	Dependent on antibody quality, relatively costly	[84]
	Chromatography (HPLC)	Separation and quantification based on hydrophobicity/size, provides purity information	Simultaneous separation, quantification, and purity check	Lower sensitivity, complex sample preparation	[85]

## Data Availability

No new data were created or analyzed in this study. Data sharing is not applicable to this article.

## Abbreviations

The following abbreviations are used in this manuscript:

FN	Fibronectin
ECM	Extracellular Matrix
EDA^+^	Extra Domain A-Containing Isoform
EDB^+^	Extra Domain B-Containing Isoform
RGD	Arg-Gly-Asp
GAGs	Glycosaminoglycans
TGF-β	Transforming Growth Factor-Beta
VEGF	Vascular Endothelial Growth Factor
MMPs	Matrix Metalloproteinases
pFN	Plasma Fibronectin
cFN	Cellular Fibronectin
PHSRN	Pro-His-Ser-Arg-Asn
IIICS	Type III Connecting Segment
LOX	Lysyl Oxidase
FA	Focal Adhesion
LINC	Linker of Nucleoskeleton and Cytoskeleton
YAP	Yes-Associated Protein
MRTF	Myocardin-Related Transcription Factor
TLR4	Toll-Like Receptor 4
NF-κB	Nuclear Factor Kappa B
PRF	Platelet-rich Fibrin
FLG	Filaggrin
LOR	Loricrin
TEWL	Transepidermal Water Loss
ROS	Reactive Oxygen Species
AGEs	Advanced Glycation End Products
SASP	Senescence-Associated Secretory Phenotype
HepII	Heparin-Binding Domain II
CBD	Collagen-Binding Domain
rhFNP	Recombinant Human Fibronectin Peptide
hMSCs	Human Mesenchymal Stem Cells
ADSCs	Adipose-Derived Stem Cells
AD-MSCs	Adipose-Derived Mesenchymal Stem Cells
PDGF-BB	Platelet-Derived Growth Factor-BB
FACSs	FN-Attached Cell Sheets
Fn-rLys-Col/SF-S	Fibronectin-Recombinant Lysostaphin-Collagen/Silk Fibroin-Sericin
rFN	Recombinant Fibronectin
SDS-PAGE	Sodium Dodecyl Sulfate-Polyacrylamide Gel Electrophoresis
WB	Western blot
IR	Infrared Spectroscopy
LC-MS/MS	Liquid Chromatography–Tandem Mass Spectrometry
BCA	Bicinchoninic Acid Assay
ELISA	Enzyme-Linked Immunosorbent Assay
HPLC	High-Performance Liquid Chromatography
EGF	Epidermal Growth Factor
PEG-FN	Polyethylene Glycol-Fibronectin
BMP-2	Bone Morphogenetic Protein-2
DAMPs	Damage-Associated Molecular Patterns
IL-8	Interleukin-8
SLE	Systemic Lupus Erythematosus
shRNA	Short Hairpin RNA
PDE4D5	Phosphodiesterase 4D5
cAMP	Cyclic Adenosine Monophosphate
FAK	Focal Adhesion Kinase
Src	Proto-oncogene Tyrosine-Protein Kinase Src
RhoA	Ras Homolog Family Member A
TAZ	Transcriptional Co-activator with PDZ-Binding Motif
QC	Quality Control
AI	Artificial Intelligence

## References

[1] da Silva P.H.R., Borges B.C., Uehara I.A., Soldi L.R., de Araújo R.A., Silva M.J.B. (2021). Chemokines and the extracellular matrix: Set of targets for tumor development and treatment. Cytokine.

[2] Huang J., Heng S., Zhang W., Liu Y., Xia T., Ji C., Zhang L.J. (2022). Dermal extracellular matrix molecules in skin development, homeostasis, wound regeneration and diseases. Semin. Cell Dev. Biol..

[3] Li W., Moretti L., Su X., Yeh C.R., Torres M.P., Barker T.H. (2024). Strain-dependent glutathionylation of fibronectin fibers impacts mechano-chemical behavior and primes an integrin switch. Nat. Commun..

[4] Dalton C.J., Lemmon C.A. (2021). Fibronectin: Molecular Structure, Fibrillar Structure and Mechanochemical Signaling. Cells.

[5] Sun Y., Hamlin A.J., Schwarzbauer J.E. (2025). Fibronectin matrix assembly at a glance. J. Cell Sci..

[6] Moretti L., Stalfort J., Barker T.H., Abebayehu D. (2022). The interplay of fibroblasts, the extracellular matrix, and inflammation in scar formation. J. Biol. Chem..

[7] Resovi A., Persichitti P., Brunelli L., Minoli L., Borsotti P., Garattini G., Tironi M., Dugnani E., Redegalli M., De Simone G. (2023). Fibronectin fragments generated by pancreatic trypsin act as endogenous inhibitors of pancreatic tumor growth. J. Exp. Clin. Cancer Res..

[8] Chen M., Hu R., Cavinato C., Zhuang Z.W., Zhang J., Yun S., Fernandez Tussy P., Singh A., Murtada S.I., Tanaka K. (2022). Fibronectin–Integrin α5 Signaling in Vascular Complications of Type 1 Diabetes. Diabetes.

[9] Dalton C.J., Dhakal S., Lemmon C.A. (2025). Measuring the biomechanical properties of cell-derived fibronectin fibrils. Biomech. Model. Mechanobiol..

[10] Su X., Ma X., Xie X., Wu H., Wang L., Feng Y., Yu Z., Liu C., Qi J., Zhu Q. (2020). FN-EDA mediates angiogenesis of hepatic fibrosis via integrin-VEGFR2 in a CD63 synergetic manner. Cell Death Discov..

[11] Dzobo K., Dandara C. (2023). The Extracellular Matrix: Its Composition, Function, Remodeling, and Role in Tumorigenesis. Biomimetics.

[12] Javid H., Oryani M.A., Rezagholinejad N., Esparham A., Tajaldini M., Karimi-Shahri M. (2024). RGD peptide in cancer targeting: Benefits, challenges, solutions, and possible integrin-RGD interactions. Cancer Med..

[13] Karbalaei M., Rezaee S.A., Farsiani H. (2020). Pichia pastoris: A highly successful expression system for optimal synthesis of heterologous proteins. J. Cell. Physiol..

[14] Jiang R., Yuan S., Zhou Y., Wei Y., Li F., Wang M., Chen B., Yu H. (2024). Strategies to overcome the challenges of low or no expression of heterologous proteins in Escherichia coli. Biotechnol. Adv..

[15] Mariotti M., Rogowska-Wrzesinska A., Hägglund P., Davies M.J. (2021). Cross-linking and modification of fibronectin by peroxynitrous acid: Mapping and quantification of damage provides a new model for domain interactions. J. Biol. Chem..

[16] Ben Abla A., Boeuf G., Elmarjou A., Dridi C., Poirier F., Changotade S., Lutomski D., Elm’selmi A. (2021). Engineering of Bio-Adhesive Ligand Containing Recombinant RGD and PHSRN Fibronectin Cell-Binding Domains in Fusion with a Colored Multi Affinity Tag: Simple Approach for Fragment Study from Expression to Adsorption. Int. J. Mol. Sci..

[17] Legrand J.M.D., Martino M.M. (2022). Growth Factor and Cytokine Delivery Systems for Wound Healing. Cold Spring Harb. Perspect. Biol..

[18] Patten J., Wang K. (2021). Fibronectin in development and wound healing. Adv. Drug Deliv. Rev..

[19] Missirlis D., Heckmann L., Haraszti T., Spatz J.P. (2022). Fibronectin anchoring to viscoelastic poly(dimethylsiloxane) elastomers controls fibroblast mechanosensing and directional motility. Biomaterials.

[20] McKeown-Longo P.J., Higgins P.J. (2021). Hyaluronan, Transforming Growth Factor β, and Extra Domain A-Fibronectin: A Fibrotic Triad. Adv. Wound Care.

[21] Bonadio J.D., Bashiri G., Halligan P., Kegel M., Ahmed F., Wang K. (2024). Delivery technologies for therapeutic targeting of fibronectin in autoimmunity and fibrosis applications. Adv. Drug Deliv. Rev..

[22] Zhou Y., Chen T., Pan Y., Liu J. (2025). Research trends on the extracellular domain A/B of fibronectin in tumor microenvironment: Scientometric and visual analysis. Discov. Med..

[23] Liu M., Chen P., Wei B., Tan H.L., Zhao Y.X., Ai L., Li N., Jiang Y.K., Lin J., Li S.J. (2025). FN1 shapes the behavior of papillary thyroid carcinoma through alternative splicing of EDB region. Sci. Rep..

[24] Wang J., Li R., Li M., Wang C. (2021). Fibronectin and colorectal cancer: Signaling pathways and clinical implications. J. Recept. Signal Transduct. Res..

[25] Lemańska-Perek A., Adamik B. (2019). Fibronectin and its soluble EDA-FN isoform as biomarkers for inflammation and sepsis. Adv. Clin. Exp. Med..

[26] Zhou Y., Chen T., Pan Y., Liu J. (2025). Exploring the mechanism of fibronectin extra domain B in the tumor microenvironment and implications for targeted immunotherapy and diagnostics (Review). Mol. Med. Rep..

[27] Longstreth J.H., Wang K. (2024). The role of fibronectin in mediating cell migration. Am. J. Physiol. Cell Physiol..

[28] Chen L., Kashina A. (2021). Post-translational Modifications of the Protein Termini. Front. Cell Dev. Biol..

[29] Luo X., Jian W. (2023). Different roles of endothelial cell-derived fibronectin and plasma fibronectin in endothelial dysfunction. Turk. J. Med. Sci..

[30] Kozlowski M.T., Zook H.N., Chigumba D.N., Johnstone C.P., Caldera L.F., Shih H.P., Tirrell D.A., Ku H.T. (2023). A matrigel-free method for culture of pancreatic endocrine-like cells in defined protein-based hydrogels. Front. Bioeng. Biotechnol..

[31] Idborg H., Oke V. (2021). Cytokines as Biomarkers in Systemic Lupus Erythematosus: Value for Diagnosis and Drug Therapy. Int. J. Mol. Sci..

[32] Yang D.R., Wang M.Y., Zhang C.L., Wang Y. (2024). Endothelial dysfunction in vascular complications of diabetes: A comprehensive review of mechanisms and implications. Front. Endocrinol..

[33] Takeyama H., Manome Y. (2023). Serum Sialyl Fibronectin Is an Indicator of Good Prognosis in Thyroid Cancer. Cancer Diagn. Progn..

[34] Malik S., Waquar S., Idrees N., Malik A. (2024). Impending role of inflammatory markers and their specificity and sensitivity in breast cancer patients. Sci. Rep..

[35] Hall R.C., Vaidya A.M., Schiemann W.P., Pan Q., Lu Z.R. (2023). RNA-Seq Analysis of Extradomain A and Extradomain B Fibronectin as Extracellular Matrix Markers for Cancer. Cells.

[36] Di Nitto C., Ravazza D., Gilardoni E., Look T., Sun M., Prodi E., Moisoiu V., Pellegrino C., Manz M.G., Puca E. (2024). An IL-7 fusion protein targeting EDA fibronectin upregulates TCF1 on CD8+ T-cells, preferentially accumulates to neoplastic lesions, and boosts PD-1 blockade. J. Immunother. Cancer.

[37] Mokhtari R.B., Ashayeri N., Baghaie L., Sambi M., Satari K., Baluch N., Bosykh D.A., Szewczuk M.R., Chakraborty S. (2023). The Hippo Pathway Effectors YAP/TAZ-TEAD Oncoproteins as Emerging Therapeutic Targets in the Tumor Microenvironment. Cancers.

[38] Kudelova E., Smolar M., Holubekova V., Hornakova A., Dvorska D., Lucansky V., Koklesova L., Kudela E., Kubatka P. (2022). Genetic Heterogeneity, Tumor Microenvironment and Immunotherapy in Triple-Negative Breast Cancer. Int. J. Mol. Sci..

[39] Wang X., Wan W., Lu J., Liu P. (2024). Inhalable FN-binding liposomes or liposome-exosome hybrid bionic vesicles encapsulated microparticles for enhanced pulmonary fibrosis therapy. Int. J. Pharm..

[40] Chu P.H., Chen S.C., Chen H.Y., Wu C.B., Huang W.T., Chiang H.Y. (2023). Astrocyte-associated fibronectin promotes the proinflammatory phenotype of astrocytes through β1 integrin activation. Mol. Cell. Neurosci..

[41] Xu W., He M., Lu Q. (2024). Fibronectin Connecting Cell Sheet Based on Click Chemistry for Wound Repair. Adv. Sci..

[42] Liu C.T., Huang L.D., Liu K., Pang K.F., Tang H., Li T., Huang Y.P., Zhang W.Q., Wang J.J., Yin G.L. (2025). Nano-Biomimetic Fibronectin/Lysostaphin-Co-Loaded Silk Fibroin Dressing Accelerates Full-Thickness Wound Healing via ECM-Mimicking Microarchitecture and Dual-Function Modulation. Int. J. Nanomed..

[43] Gimeno-LLuch I., Benito-Jardón M., Guerrero-Barberà G., Burday N., Costell M. (2022). The Role of the Fibronectin Synergy Site for Skin Wound Healing. Cells.

[44] Hu Y., Shi H., Ma X., Xia T., Wu Y., Chen L., Ren Z., Lei L., Jiang J., Wang J. (2023). Highly stable fibronectin-mimetic-peptide-based supramolecular hydrogel to accelerate corneal wound healing. Acta. Biomater..

[45] Setiawati A., Jang D., Cho D., Cho S., Jeong H., Park S., Gwak J., Ryu S.R., Jung W.H., Ju B.G. (2021). An Accelerated Wound-Healing Surgical Suture Engineered with an Extracellular Matrix. Adv. Healthc. Mater..

[46] Zhang Y., Li Z., Wang J., Zhang W., Li J., Liu Y., Wang Y., Zhang X., Li H., Chen X. (2024). A Bioactive Fibronectin-Functionalized Surgical Mesh with Dual-Antimicrobial and Pro-Healing Properties for Abdominal Wall Reconstruction. ACS Biomater. Sci. Eng..

[47] Zhang W., Akhtar N., Zhao J., Spandau D.F., Kaplan M.H. (2024). Fibronectin Promotes Wound Healing in an Atopic Human Skin Xenografting Model. J. Investig. Dermatol..

[48] Wang L., Smith J., Johnson A.B., Davis R., Brown K.L., Wilson M.P. (2023). Development of a Fibronectin-Enriched Collagen Sponge for Chronic Wound Treatment: Efficacy in a Diabetic Mouse Model. J. Tissue Eng. Regen. Med..

[49] Chen H., Zhou M., Li F., Wu S., Xu J., Thompson C., Williams R. (2025). Enhancing Diabetic Wound Healing with a Fibronectin-Mimetic Peptide Hydrogel: Synergistic Effects of Angiogenesis and ECM Remodeling. Pharmaceutics.

[50] Li X., Mao X., Cong J., Zhang Q., Chen W., Yan K., Huang Y., Su D., Xiang Q. (2024). Recombinantly expressed rhFEB remodeled the skin defect of db/db mice. Appl. Microbiol. Biotechnol..

[51] Cai X., Zhu J., Luo X., Jin G., Huang Y., Li L. (2023). A Thermally Stable Recombinant Human Fibronectin Peptide-Fused Protein (rhFN3C) for Faster Aphthous Ulcer (AU) Healing. Bioengineering.

[52] Cong J., Cheng Y., Liu T., Cai X., Xu J., Guo R., He R., Xiang Q. (2025). Recombinant human fibronectin segment (rhFN1024) hydrogel carried hPDLSCs to repair diabetic trauma by activated NF-κB signaling pathway. Regen. Biomater..

[53] Liu X.P., Liu C.L., Xie J., Li T.H., Li H., Bai X.J., Li Z.F., W W. (2021). Clinical application of platelet-rich fibrin in chronic refractory wounds. J. Clin. Surg..

[54] Shi J.Y., Cui Z.J., Zhao H. (2023). The research on clinical use of platelet-rich fibrin in treatment of chronic wounds. J. Clin. Surg..

[55] Lin Z., Nica C., Sculean A., Asparuhova M.B. (2021). Positive Effects of Three-Dimensional Collagen-Based Matrices on the Behavior of Osteoprogenitors. Front. Bioeng. Biotechnol..

[56] Casanova M.R., Reis R.L., Martins A., Neves N.M. (2020). Fibronectin Bound to a Fibrous Substrate Has Chondrogenic Induction Properties. Biomacromolecules.

[57] Chen Y., Fan Y., Dong Y., Yu X., Gao J., Ma X. (2025). Therapeutic Effect of a Recombinant Human Fibronectin Construct in Skeletal Muscle Repair and Oxidative Stress. Int. J. Mol. Sci..

[58] Hong Y., Xin J., Wang P., Song Y., Fan X., Yang L., Guo G., Fu D., Dai Y., Zhang F. (2025). Enhancing the biocompatibility of phakic intraocular lens via selective fibronectin trapping. Acta. Biomater..

[59] Li Z., Bratlie K.M. (2021). Macrophage Phenotypic Changes on FN-Coated Physical Gradient Hydrogels. ACS Appl. Bio. Mater..

[60] Wacker M., Riedel J., Walles H., Groll J., Lübberstedt M., Zimmermann S., Rottmar M., Kania G., Rieger M.A. (2021). Comparative Evaluation on Impacts of Fibronectin, Heparin-Chitosan, and Albumin Coating of Bacterial Nanocellulose Small-Diameter Vascular Grafts on Endothelialization In Vitro. Nanomaterials.

[61] Zou J., Chen L.C., Wu Y.Q., Ye Z.H. (2021). Research Progress of Fibronectin and Its Application in Cosmetics. Deterg. Cosmet..

[62] Farage M.A. (2019). The Prevalence of Sensitive Skin. Front. Med..

[63] Kim Y., Lim K.M. (2021). Skin barrier dysfunction and filaggrin. Arch. Pharmacal Res..

[64] Wang Y., Zhang Q., Liao H., Lu X., Cong J., Liu Z., Liu Q., Deng C., Cheng Y., Shu P. (2025). Anti photoaging mechanism of a novel recombinant human fibronectin peptide (rhFNP) derived from the extracellular matrix. Heliyon.

[65] Wang J., Li Y.H., Liu T.Z., Zhao W.B. (2025). Fibronectin-based Scalp Repair Products and Their Applications. Flavour Fragr. Cosmet..

[66] Xie Z., Wang X., Ren X., Ge X. (2024). MMP12 disrupts epithelial barrier integrity in oral lichen planus by degrading fibronectin. Sci. Rep..

[67] Dong Y., Zhu W., Lei X., Luo X., Xiang Q., Zhu X., Pan Q., Jin P., Cheng B. (2022). Treatment of Acute Wounds with Recombinant Human-Like Collagen and Recombinant Human-Like Fibronectin in C57BL/6 Mice Individually or in Combination. Front. Bioeng. Biotechnol..

[68] Papaccio F., D Arino A., Caputo S., Bellei B. (2022). Focus on the Contribution of Oxidative Stress in Skin Aging. Antioxidants.

[69] Levi N., Papismadov N., Solomonov I., Sagi I., Krizhanovsky V. (2020). The ECM path of senescence in aging: Components and modifiers. FEBS J..

[70] Shmulevich R., Krizhanovsky V. (2021). Cell Senescence, DNA Damage, and Metabolism. Antioxid. Redox Signal..

[71] Papaccio G., Park B.H., Jeong E.S., Lee S., Jang J.H. (2021). Bio-functionalization and in-vitro evaluation of titanium surface with recombinant fibronectin and elastin fragment in human mesenchymal stem cell. PLoS ONE.

[72] Guillem-Marti J., Gelabert M., Heras-Parets A., Pegueroles M., Ginebra M.P., Manero J.M. (2019). RGD Mutation of the Heparin Binding II Fragment of Fibronectin for Guiding Mesenchymal Stem Cell Behavior on Titanium Surfaces. ACS Appl. Mater. Interfaces.

[73] Tragoonlugkana P., Pruksapong C., Ontong P., Kamprom W., Supokawej A. (2024). Fibronectin and vitronectin alleviate adipose-derived stem cells senescence during long-term culture through the AKT/MDM2/P53 pathway. Sci. Rep..

[74] Chen L., Carlton M., Chen X., Kaur N., Ryan H., Parker T.J., Lin Z., Xiao Y., Zhou Y. (2021). Effect of fibronectin, FGF-2, and BMP4 in the stemness maintenance of BMSCs and the metabolic and proteomic cues involved. Stem Cell Res. Ther..

[75] Lin X., Filppula A.M., Zhao Y., Shang L., Zhang H. (2025). Mechanically regulated microcarriers with stem cell loading for skin photoaging therapy. Bioact. Mater..

[76] Dyakov I.N., Mavletova D.A., Chernyshova I.N., Snegireva N.A., Gavrilova M.V., Bushkova K.K., Dyachkova M.S., Alekseeva M.G., Danilenko V.N. (2020). FN3 protein fragment containing two type III fibronectin domains from B. longum GT15 binds to human tumor necrosis factor alpha in vitro. Anaerobe.

[77] Saunders J.T., Schwarzbauer J.E., Ginsberg M.H. (2019). Fibronectin matrix as a scaffold for procollagen proteinase binding and collagen processing. Mol. Biol. Cell.

[78] Luo X., Geng D., Zhang Q., Ye T., Zhang Y., Li Z., Huang Y., Xiang Q. (2022). Recombinant expression a novel fibronectin—Collage fusion peptide modulating stem cell stemness via integrin β3. Appl. Microbiol. Biotechnol..

[79] Kielkopf C.L., Bauer W., Urbatsch I.L. (2021). Sodium Dodecyl Sulfate-Polyacrylamide Gel Electrophoresis of Proteins. Cold Spring Harb. Protoc..

[80] Liu L.L., Wang L., Zonderman J., Rouse J.C., Kim H.Y. (2020). Automated, High-Throughput Infrared Spectroscopy for Secondary Structure Analysis of Protein Biopharmaceuticals. J. Pharm. Sci..

[81] Alley W.R., Mann B.F., Novotny M.V. (2013). High-sensitivity analytical approaches for the structural characterization of glycoproteins. Chem. Rev..

[82] Højrup P. (2024). Analysis of Polypeptides by Amino Acid Analysis. Methods Mol. Biol..

[83] Cohen L., Walt D.R. (2019). Highly Sensitive and Multiplexed Protein Measurements. Chem. Rev..

[84] Liu J., Yang G., Gao X., Zhang Z., Liu Y., Liu Q., Chatel J.-M., Jiang Y., Wang C. (2019). Recombinant invasive Lactobacillus plantarum expressing fibronectin binding protein A induce specific humoral immune response by stimulating differentiation of dendritic cells. Benef. Microbes.

[85] Liu Y., Gao J., Liu L., Kang J., Luo X., Kong Y., Zhang G. (2022). Identification and Characterization of Fibronectin-Binding Peptides in Gelatin. Polymers.

[86] Richardson S.A., Rawlings T.M., Muter J., Walker M., Brosens J.J., Cameron N.R., Eissa A.M. (2018). Covalent Attachment of Fibronectin onto Emulsion-Templated Porous Polymer Scaffolds Enhances Human Endometrial Stromal Cell Adhesion, Infiltration, and Function. Macromol. Biosci..

